# Biology and Clinical Relevance of HCMV-Associated Adaptive NK Cells

**DOI:** 10.3389/fimmu.2022.830396

**Published:** 2022-03-31

**Authors:** Fei Gao, Zhengwei Zhou, Ying Lin, Guang Shu, Gang Yin, Tianxiang Zhang

**Affiliations:** ^1^ Immuno-Oncology Laboratory, Department of Pathology, School of Basic Medicine, Central South University, Changsha, China; ^2^ Department of Immunobiology, Yale University School of Medicine, New Haven, CT, United States

**Keywords:** HCMV, adaptive NK cells, ADCC, epigenetic remodeling, therapeutic potential

## Abstract

Natural killer (NK) cells are an important component of the innate immune system due to their strong ability to kill virally infected or transformed cells without prior exposure to the antigen (Ag). However, the biology of human NK (hNK) cells has largely remained elusive. Recent advances have characterized several novel hNK subsets. Among them, adaptive NK cells demonstrate an intriguing specialized antibody (Ab)-dependent response and several adaptive immune features. Most adaptive NK cells express a higher level of NKG2C but lack an intracellular signaling adaptor, FcϵRIγ (hereafter abbreviated as FcRγ). The specific expression pattern of these genes, with other signature genes, is the result of a specific epigenetic modification. The expansion of adaptive NK cells *in vivo* has been documented in various viral infections, while the frequency of adaptive NK cells among peripheral blood mononuclear cells correlates with improved prognosis of monoclonal Ab treatment against leukemia. This review summarizes the discovery and signature phenotype of adaptive NK cells. We also discuss the reported association between adaptive NK cells and pathological conditions. Finally, we briefly highlight the application of adaptive NK cells in adoptive cell therapy against cancer.

## 1 Introduction

As a crucial member of the innate lymphoid cell (ILC) family, natural killer (NK) cells specialize in killing damaged, infected, or transformed cells and releasing cytokines as immune modulators (1, 2). Unlike adaptive immune cells, NK cells do not need to be exposed to the antigen (Ag) in advance to mature effector functions. Therefore, NK cells are considered at the forefront of the host immune system. The important role of NK cells in restricting viral infection or tumor metastasis has been observed *in vivo* in both murine and human studies (3, 4). However, although a series of stochastically expressed activating and inhibitory receptors have been described, the recognition of NK and target cells is far less clear (5). The final activity of NK cells depends on the balance between activating and inhibitory signals transduced by these receptors.

Accumulating studies have shown that NK cells demonstrate adaptive immune features, such as clonal expansion and immune memory, resulting in a diversely functional repertoire and stronger responses to previously encountered stimuli (6). For example, murine Ly49H-dependent NK cells are capable of responding specifically to *murine cytomegalovirus* (MCMV) and mount stronger reactions to secondary challenges (7, 8). Moreover, murine hepatic CXCR6^+^ NK cells mediate memory response to a secondary challenge to hapten or viruses in the absence of T and B cells (9–11).

**Figure 1:**
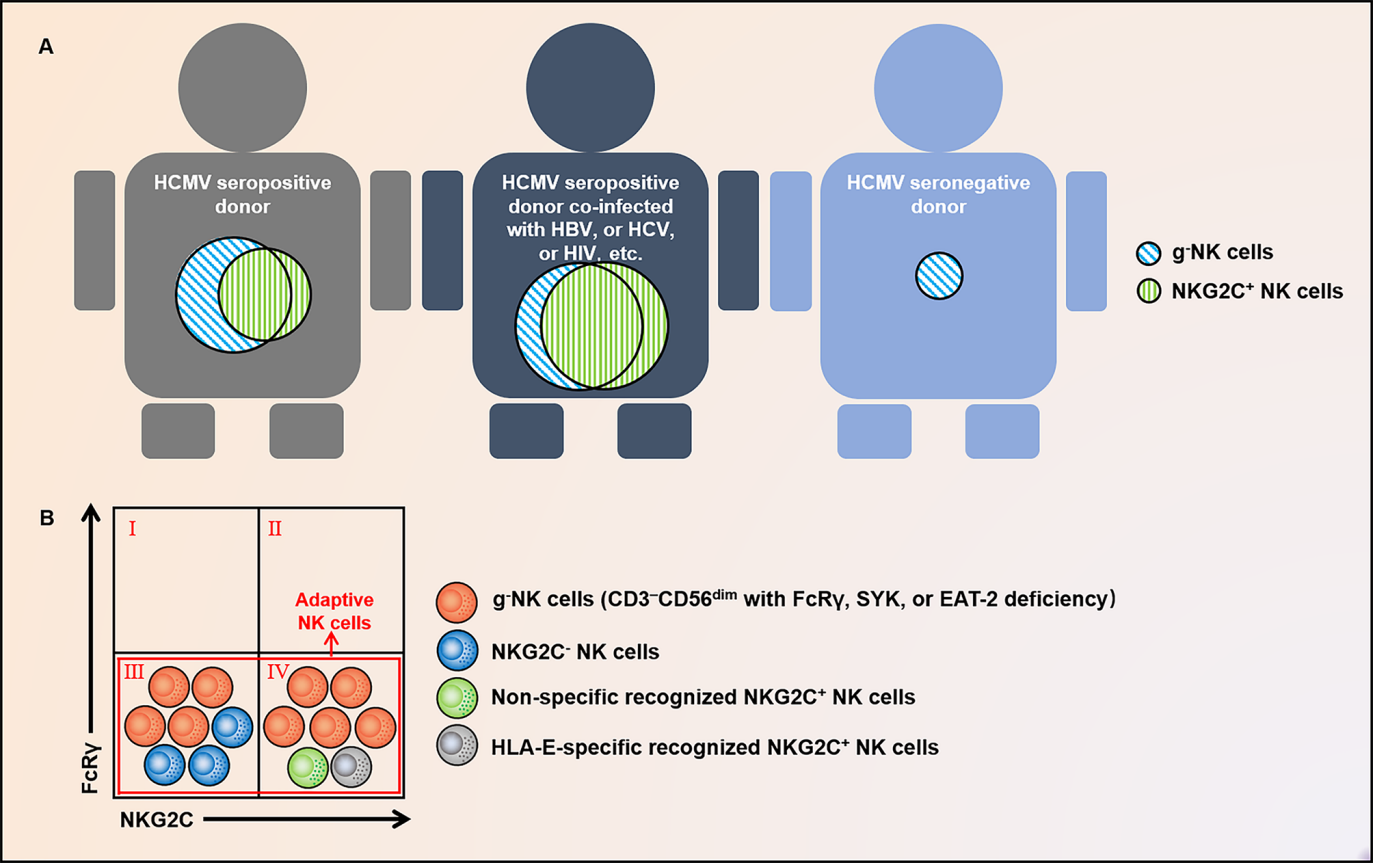
Distribution and relationship of g^-^NK and NKG2C^+^ NK cells. **(A)** g^-^NK cells persist and expand long-term in healthy individuals with prior HCMV infection, and their proportion will increase under the co-infection of HBV, HCV, HIV, and other chronic viruses. Exceptionally, a fraction of this subset has also been observed in HCMV^-^ individuals. **(B)** g^-^NK cells characterized by FcRγ deficiency are different from NK cells marked by NKG2C and are composed of both NKG2C^+^ and NKG2C^-^ subsets. In addition to this subset, adaptive NK cells comprise a subset of non-specifically recognized and HLA-E-specifically recognized NKG2C^+^ NK cells. These subgroups largely intersect. g^-^NK, γ chain-negative natural killer; HCMV, human cytomegalovirus; HBV, hepatitis B virus; HCV, hepatitis C virus; HIV, human immunodeficiency virus; FcϵRIγ or FcRγ, IgE receptor subunit-γ; EAT2, EWS/FLI1-activated transcript 2; SYK, spleen tyrosine kinase; and HLA-E, HLA class I histocompatibility antigen; alpha chain E.

The adaptive features of human NK (hNK) cells have been extensively studied during the last decade. Human cytomegalovirus (HCMV), an enveloped member of the Herpesviridae family, has been shown to extensively alter the phenotype and function of hNK cells, as reflected by a strong association with an NK subset expressing NKG2C, a receptor for HLA class I histocompatibility antigen, alpha chain E (HLA-E) (12, 13). Of interest, these NK cells specifically respond to HCMV peptides presented by HLA-E (14). Subsequent studies have also suggested that HCMV infection is positively associated with a group of peripheral blood (PB) NK cells, g^-^NK, characterized by the absence of FcRγ, a signaling adaptor protein. NKG2C^+^ NK and g^-^NK subsets are largely overlapping but not identical, and g^-^NK cells also demonstrate adaptive features, such as clonal expansion, augmented effector function, and extended lifespan (15–17) (**Figure 1**). In 2015, hNK cells lacking FcRγ, spleen tyrosine kinase (SYK), and EWS/FLI1-activated transcript 2 (EAT-2), along with silencing of promyelocytic leukemia zinc finger (PLZF) expression, were termed “adaptive NK cells” by Schlums et al. (18). A subset of NKG2C^+^ adaptive NK cells can specifically recognize HCMV strains encoding variable UL40 peptides and can further expand and differentiate in response to stimulation by pro-inflammatory factors (14). However, available evidence suggests that the above-mentioned recognition of NKG2C with HLA-E plus peptides is not observed in NKG2C^-^ adaptive NK cells (19). Immunological memory is defined as the enhanced response to the re-challenge of the same Ag, and immunological memory cells can persist for years, even for a lifetime (20). Thus far, there are no data indicating that adaptive NK cells can recognize various Ags and mount an enhanced response. Rather, adaptive NK cells achieve a broader spectrum of Ag specificity through antibody (Ab)-dependent function. NK cells have long been considered short-lived innate effector cells. However, several lines of evidence indicate that adaptive NK cells have an unexpectedly long lifespan compared to conventional NK (c-NK) cells. Resembling adaptive memory cells, adaptive NK cells can live for months to years (16). Additionally, adaptive NK cells have been found to expand in response to other viral infections, such as hepatitis B virus (HBV), hepatitis C virus (HCV), human immunodeficiency virus (HIV), herpes simplex virus (HSV), and influenza virus (21–25). This review summarizes the research progress of adaptive NK cells, with a special focus on molecular hallmarks, epigenetic regulation, differentiation, and tissue distribution. Additionally, we focus on the correlation between adaptive NK and multiple clinical diseases, such as viral infection and cancer, and put forward prospects that target them to improve immunotherapy.

## 2 Discovery and Molecular Markers of Human Adaptive NK Cells

The discovery of human adaptive NK cells can be traced back to more than 1 decade ago (12, 15, 16, 18, 26–30). The expansion of a CD94/NKG2C^+^ NK subset in the PB of HCMV^+^ donors was first described by Guma et al. in 2004 (12). These NK cells express lower levels of NKp30 and NKp46 compared to CD94/NKG2A^+^ NK cells isolated from the same donors. The expansion of CD94/NKG2C^+^ NK cells is associated only with HCMV but not with HSV or EBV serology (12). In 2011, Lopez-Verges et al. described an expansion of CD57^+^NKG2C^hi^ NK cells in bone marrow (BM) transplantation recipients with acute HCMV infection (26). In 2012, Foley et al. suggested that these HCMV-induced NKG2C^+^ NK cells were transplantable and expanded in response to recipient CMV Ag *in vivo*, thus demonstrating memory-like phenotype (27). Earlier, in the same year, Hwang et al. documented a group of FcRγ-deficient NK cells, g^-^NK, in the PB of one-third of healthy donors (15). These cells are considered likely to arise from clonal expansion, express low levels of NKp46 and NKp30, and respond poorly to K562 cells. However, g^-^NK cells express an almost normal level of CD16 and demonstrate an enhanced response to CD16 cross-linking. The association between the presence of g^-^NK cells and HCMV infection was described by Zhang, T. et al. in 2013 (16). In the same year, Beziat et al. studied the role of both self-specific inhibitory killer cell immunoglobulin-like receptors (KIRs) and activating KIRs during clonal expansion of NK cells in response to CMV infection (28). The association of FcRγ and NKG2C expression was finally described by Schlums et al. and Lee et al. in 2015 (18, 29) and further discussed by Muntasell et al. in 2016 (30). Additionally, HCMV-associated NKG2C^+^ NK cells were named adaptive NK cells by Muntasell et al. (30). As numerous researchers have considered NKG2C^+^ NK cells that have previously encountered HCMV as or even equal to adaptive NK cells, such generalization may ignore subsets of cells including NKG2C^-^ g^-^NK cells (29); we hereby find a legible explanation. A study by Liu, L.L. et al. found that a characteristic footprint of adaptive NK cells existed in NKG2C^-/-^ donors, including terminal differentiation phenotypes, functional reprogramming, and epigenetic remodeling of the interferon-gamma (IFN-γ) promoter (19).

Detailed studies have shown that HCMV-associated NK cells undergo extensive epigenetic modulation compared to c-NK cells. The defect in FcRγ is not due to gene mutation but due to hypermethylation of the *FCER1G* promoter. Epigenetic modification is also the underlying mechanism controlling a specified function. Compared with c-NK cells, adaptive NK cells have a superior ability to produce IFN-γ and tumor necrosis factor alpha (TNF-α) in response to stimuli through CD16. Consistently, *IFNG* and *TNF*, encoding IFN-γ and TNF-α, respectively, show hypomethylation at the gene loci, highlighting the mechanism of the elevated capacity of adaptive NK cells to release these cytokines following activation *via* CD16 and NKG2C (31, 32).

**Figure 2:**
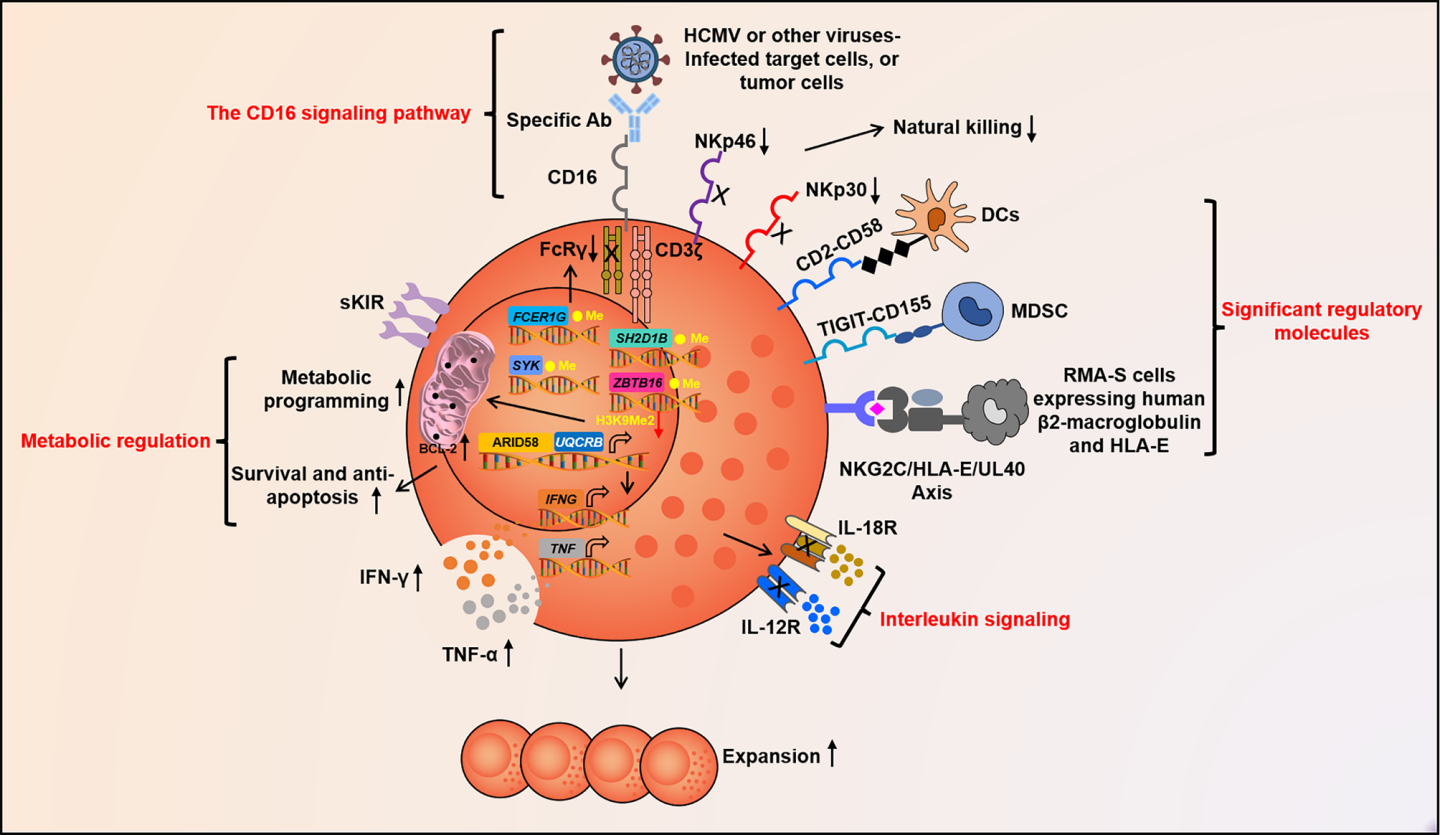
Molecular factors govern adaptive NK cell function. Fc segment of Abs can take shape specific signal of adaptive NK cells to recognize target cells by binding to CD16. NKG2C, CD2, TIGIT, and other unknown molecules may act as crucial signaling pathway for the activation or inhibition of adaptive NK cells. IL-12, IL-18, and other cytokines together constitute the pro-inflammatory cytokine signal, which guides adaptive NK cell differentiation, expansion, and activation. Epigenetic programming promotes stochastic DNA methylation-dependent allelic silencing of *FCER1G*, *SYK*, and *SH2D1B*. These ultimately contribute to *IFNG* promoter hypomethylation and format heterogeneous adaptive NK cell repertoire. The up-regulation of ARID5B and its inducing *UQCRB* with low H3K9Me2 in the promoter region alter metabolic reprogramming, prolong survival, and motivate abundant IFN-γ secretion of adaptive NK cells. Abs, antibodies; CD, cluster of differentiation; TIGIT, T-cell immunoglobulin and ITIM domain; interleukin, IL; IFN-γ, Interferon-gamma; TNF-α, Tumor necrosis factor alpha; and ARID5B, AT-rich interaction domain 5.

Though adaptive NK cells exhibit a stronger response to CD16 cross-linking, the expression of several signaling molecules of the CD16 pathway, including ZAP70, PLCγ1, PLCγ2, LCP2, and PIK3CA, is not significantly different between adaptive NK and c-NK cells. Of interest, SYK expression is absent in some adaptive NK cells due to promoter hypermethylation. The SYK-deficiency is closely related to HCMV infection, rather than HSV-1 or HSV-2, and can be passed along to daughter cells. The SYK-deficiency in adaptive NK cells does not impair their function but is associated with enhanced responsiveness to CD16 cross-linking. Notably, SYK-deficient adaptive NK cells have mainly been found among g^-^NK cells (18, 29). Another epigenetic signature of adaptive NK cells is the hypermethylation of the promoters of several important transcription factor (TF)-encoding genes, including *ZBTB16* (encoding PLZF), *SH2D1B* (encoding EAT-2), and *DAB2* (encoding DAB2). *ZBTB16* silencing results in reduced expression of PLZF and deficiency of IL-12Rβ1 and IL-18Rα. Indeed, adaptive NK cells demonstrate a marginal response to IL-12 and IL-18 stimulation (18) (**Figure 2**).

It has been shown that only NKG2C^hi^ NK cells are expanded in HCMV seropositive individuals and experience epigenetic remodeling of the conserved non-coding sequence 1 (*CNS1*) of *IFNG*, similar to memory CD8^+^ T or Th1 cells. To cater to the engagement of NKG2C and 2B4, the accessibility of *CNS1* is necessary to increase *IFNG* transcription. Indeed, a closed configuration of *CNS1* has been detected in NKG2C^-^ NK cells from HCMV^+/-^ individuals and in NKG2C^+^ NK cells from HCMV^-^ individuals. Remarkably, among HCMV^+^ individuals, the open configuration of CNS1 occurs exclusively in expanded NKG2C^hi^ self-MHC specific KIRs (sKIRs)^+^ NK cells. Consistently, NKG2C^+^ NK cells from HCMV^+^ individuals do not show a closed configuration of *CNS1*, similar to NKG2C^-^ NK cells. The *CNS1* methylation that occurs in expanded NKG2C^hi^ NK cells does not rely on the expression of sKIRs or CD57 and is stably retained in the daughter cells (31). Taken together, epigenetic modification is responsible for specific phenotypes of adaptive NK cells (**Figure 2**).

Collectively, adaptive NK cells have the following characteristics: 1) associated with prior HMCV infection; 2) lack expression of FcRγ, tyrosine kinase SYK, signaling molecules EAT-2 and DAB2, and TF PLZF; 3) do not necessarily express NKG2C or CD57; 4) express sKIRs but lack NKG2A; 5) down-regulation of natural cytotoxic receptors (NCRs) (i.e., NKp46 and NKp30), CD161, Siglec7, ITGA6, CD7, PECAM1, and TIM-3, and lack of IL-12R and IL-18R; 6) stable surface level of NKG2D and CD16, and up-regulation of ILT2, CD2, FAS, CD11a, CD44, CCR5, and Bcl-2; 7) mount long-term effective recall response directed by Fc receptor (FcR)-mediated Ab-dependent target recognition, possibly *via* specific recognition by NKG2C or other known or unknown receptor; 8) epigenetic imprinting; and 9) Ab-dependent enhanced functional capabilities, including Ab-dependent cell-mediated cytotoxicity (ADCC), *in vitro* expansion, and cytokine production (15, 16, 18, 29, 33, 34).

## 3 Functional Characteristics of Adaptive NK Cells

### 3.1 Expansion and Effector Function

The detailed mechanism mediating adaptive NK cell proliferation remains elusive, though NKG2C^+^ NK cell expansion can be observed both *in vitro* and *in vivo* (35). A similar response to different viral infections indicates that cellular factors mediate this expansion phenotype. Indeed, existing evidence suggests that IL-12, IL-15, and HLA-E are critical for the expansion of NKG2C^+^ NK cells (36–38). Consistently, HLA-E and inflammatory cytokines can be up-regulated during HCMV, hantavirus, or HIV infection. The involvement of cytokines in mediating NKG2C^+^ NK cell expansion is also supported by *in vivo* observations in humans infected with viruses. Up-regulated IL-12 and IL-15 are positively associated with HCMV serostatus in children (39). Moreover, IL-12Rβ1 deficiency impairs the generation of NK cells with adaptive features (40). However, the role of IL-12 in mediating the expansion of adaptive NK cells requires further study, given their low IL-12 receptor expression and marginal response to IL-12 plus IL-18 stimulation *in vitro* (18, 29). Of interest, adaptive NK cells show clonal expression of inhibitory KIRs specific for self-HLA class I molecules, suggesting that licensing is involved in adaptive NK cell generation or expansion (15, 18, 28). Notably, KIRs on adaptive NK cells tend to be ligands of HLA-C1/C2, which are also ligands for stimulating KIRs (such as 2DS4), which are correlated with resistance to viral infection (28, 41). Whether KIRs play a role in adaptive NK cell expansion requires further study.

CD16 is a low-affinity Fc receptor that is mainly expressed by NK cells but can also be detected on neutrophils and monocytes. The intracellular part of CD16 is short and, as a result, has a limited ability to transduce signals in isolation. CD16 couples with CD3ζ and FcRγ, to deliver an activating signal. Though many adaptive NK cells, mostly g^-^NK, do not express FcRγ, their response to CD16 cross-linking is even better than other NK cells that express both CD3ζ and FcRγ. While the underlying mechanism for this phenotype requires further study, it has been shown that knockout of FcRγ by CRISPR-Cas9-mediated gene editing can reconstitute this phenotype in c-NK cells (42). This finding is interesting because it indicates, for the first time, that FcRγ can inhibit the CD16 pathway of hNK cells. It also suggests that ADCC function can be enhanced by manipulating a single gene other than CD16 itself. FcRγ only contains one ITAM motif, while CD3ζ contains three. The simplest explanation is that the loss of FcRγ makes CD16 more available to CD3ζ, although the molecular mechanism of this finding warrants further study. Upon MCMV infection, Ly49H, through multiple surface expression, binds to m157 to drive the differential expansion of memory NK cell clones, such as TCR affinity. Consistent with experiments on mice that have reported that the expression of Ly49H has a fairly strong correlation with Ly49H^+^ NK cell expansion, a positive correlation between the expression of NKG2C and the expansion of NKG2C^+^ cells has also been observed in HCMV-seropositive donors (43, 44). However, such studies have not been reported for g^-^NK cells that are not specifically recognized *via* NKG2C, suggesting that the specifically activated signal CD16-Fc segment or some other unknown Ag recognition pathway is involved in avidity selection and expansion. Remarkably, the enhanced Ab-dependent response of g^-^NK cells is not limited to HCMV-infected target cells and also applies to HSV-1-infected target cells; this indicates that g^-^NK cells have considerable potential to mediate Ab-dependent cross-protection against broad-spectrum viral infections (16). In HCMV^+^ individuals, irrespective of NKG2C^+^ or NKG2C^-^, HCMV-specific Ab invariably induces an increase in IFN-γ and TNF-α production by g^-^NK cells, suggesting that the CD16 signaling pathway may be the crucial signal for adaptive NK cell responses (29). Notably, Lee et al. reported that g^-^NK cells can be significantly expanded *in vitro* following the application of autologous HCMV^+^ serum. It is unclear whether this expansion mainly relies on the CD16 pathway or whether other cytokines are also involved (29) (**Figure 2**).

NK cells are immune sentinels that eradicate target cells and release various cytokines and chemokines to tune the adaptive immune response (45). The activation of NK cells is determined by the balance between activating and inhibitory signals. NCRs, such as NKp30, NKp46, and NKG2D, are well-known to be important for natural killing activity, while CD16 mediates ADCC (46). A large portion of adaptive NK cells, mostly g^-^NK, have a limited number of membrane NCRs and therefore only show a marginal response when co-cultured with K562 cells (15).

### 3.2 Significant Regulatory Molecules

#### 3.2.1 NKG2C

Human *NKG2C*, also known as *KLRC2*, is located at 12p13 in the NK complex (47). NKG2C binds to HLA-E and transmits an activating signal to NK cells. HCMV-encoded UL40 peptide can stabilize HLA-E on the surface of HCMV-infected cells and affect HLA-E presentation and binding to NKG2C (48). Given that NKG2C is a critical marker for adaptive NK cells, its role in regulating the function of adaptive NK cells has been well investigated. Interesting progress has been reported by Hammer et al., who demonstrated that UL40-encoded peptide-pulsed HLA-E-expressing cells can trigger an elevated frequency and number of NKG2C^+^ adaptive NK cells. Notably, due to the variability of the peptide, its ability to induce IFN-γ, TNF-α, and CCL3 secretion by NKG2C^+^ adaptive NK cells differs in the order of VMAPRTLFL > VMAPRTLIL > VMAPRTLVL, and the same phenomenon is also observed in the cytotoxicity assay (14). Merino et al. stimulated NKG2C chronically *via* anti-NKG2C and found a significant proliferation of adaptive NK cells with epigenetic remodeling similar to that of exhausted CD8^+^ T cells. Of note, this chronic stimulation also drives the expression of lymphocyte-activation gene 3 (LAG3), programmed death-1 (PD-1), and T-cell immunoglobulin and ITIM domain (TIGIT), which confer the phenotype of exhausted T cells (49).

In a healthy population, the NKG2C haplotype can be detected in approximately 20% of donors, while homozygous deletion is common (50). The *NKG2C* copy number not only determines the surface expression level of NKG2C receptor but also directly contributes to the abundance, differentiation, and distribution of adaptive NK cell subset in response to HCMV, as demonstrated by NKG2C^+/del^ compared to NKG2C^+/+^ healthy individuals (30).

NK cells in NKG2C homozygous and hemizygous subjects show differences in response to NKG2C signals (i.e., iCa^2+^ influx), degranulation, and IL-15-dependent proliferation, further indicating that the receptor is involved in forming the HCMV-induced reconfiguration of the NK-cell compartment (51). Of note, the g^-^NK subset is composed of both NKG2C^+^ and NKG2C^-^ NK cells. Lee et al. found that g^-^NK cells could respond to HCMV-infected target cells, regardless of NKG2C expression, in the presence of HCMV^+^ plasma (29). Liu, L.L. et al. made a similar observation in NKG2C^-/-^ donors (19) (**Figure 2**). However, the frequency of NKG2C^+^ NK cells after g^-^NK expansion has not been well characterized. NKG2C^-^ g^-^NK cells can be expanded *in vitro* (unpublished data), indicating that pathways other than NKG2C can mediate adaptive NK cell expansion.

#### 3.2.2 CD2

Glycoprotein CD2 is a co-stimulatory receptor expressed mainly on T and NK cells, which binds to LFA3/CD58, a cell surface protein expressed on other cells, such as dendritic or endothelial cells. Previous studies have shown that CD2 is involved in the formation of immune synapses (ISs) between immune cells and Ag presenting cells and is up-regulated on memory and activated T cells. Similarly, the presence of CD2 has been observed in the ISs formed between NK and target cells. CD2 may interact with CD16 in cis to promote its reposition as a linkage between CD16 and the actin cytoskeleton (52). CD2 expression is significantly increased in adaptive NK cells of NKG2C^+^ and NKG2C^-/-^ donors. Additionally, the interaction between CD2 and CD16 enhances the Ab-mediated response in adaptive NK cells. Co-ligation of CD2 and CD16 leads to significantly higher levels of phosphorylation of all signaling molecules, including CD3-ζ, ZAP70/SYK, SLP76, LAT, ERK1/2, and S6RP, suggesting that CD16 and CD2 synergistically activate the MAP kinase and mTOR pathways (19) (**Figure 2**).

#### 3.2.3 TIGIT

TIGIT is a momentous inhibitory molecule in the PVR/nectin family and is mainly expressed on T and NK cells, and its immune-modulatory function has been studied in the context of autoimmunity, viral immunity, and cancer (53). Ligating TIGIT with high-affinity CD155 and low-affinity CD112 can recruit SH2 domain-containing inositol-5-phosphatase (SHIP) to ITIM and inhibit the activation of T and NK cells. Indeed, tumor-infiltrating NK cells are found to express high levels of TIGIT. Blocking Abs that target TIGIT and CD155 interaction could up-regulate NK functions and inhibit tumor growth in a mouse model (54). PB adaptive NK cells express low levels of TIGIT and, therefore, in contrast to c-NK cells, are less sensitive to TIGIT pathway inhibition mediated by myeloid-derived inhibitory cells (MDSCs) (55) (**Figure 2**).

### 3.3 Metabolic Regulation

An isoform of AT-rich interaction domain 5 (ARID5B) is selectively up-regulated and involved in supporting mitochondrial membrane potential, the expression of electron transport chain (ETC) components, oxidative metabolism, survival, and IFN-γ production in adaptive NK cells. The increased metabolism observed in this subset appears to depend, at least in part, on the up-regulated expression of ARID5B and the induction of genes encoding components of ETC, including the ETC complex III gene *UQCRB*. Synchronously, a significantly inferior H3K9Me2 enrichment at the *UQCRB* promoter was observed. Beyond this, the decrease in ARID5B is associated with increased apoptosis and decreased expression of BCL-2, which suggests that ARID5B is essential for the survival of adaptive NK cells. As BCL-2 is located in mitochondria to counteract the production of ROS, a byproduct of ETC activity, increased BCL-2 in adaptive NK cells may be crucial to limit oxidative stress (56) (**Figure 2**). Taken together, enhancing ARID5B and mitochondrial function is a promising strategy to improve cell survival in NK cell-mediated immunotherapy.

### 3.4 Interleukin Signaling Regulation

The common gamma chain (γc) receptor family cytokines, including IL-2, IL-15, and IL-21, are required for the generation, persistence, and homeostasis of adaptive NK cells (57). IL-2 was first discovered as a T cell growth factor in 1976 and was later found to have various pleiotropic properties, including the ability to augment the cytolytic activities of NK cells and cytotoxic T cells. The IL-2 receptor α chain (IL-2Rα) binds IL-2 at intermediate-affinity (K_d_ ~ 10^−9^ M) but can combine with IL-2Rβ and IL-2Rγ to form a heterotrimer with a much higher affinity. Equally important, NK cells cannot survive for an extended time without IL-15 signaling, as IL-15 promotes the continuous expression of the key anti-apoptotic protein MCL-1, and adaptive NK cells are no exception. Moreover, IL-2 and IL-15 play a key role in NK cell function through the JAK1/3 and STAT5-dependent signaling pathway, which can bind to the IL-2R complex or IL-15Rβ/α on the surface of NK cells to induce the activation of β/γ-related JAK1 and JAK3 tyrosine kinases. This phosphorylation further leads to the recruitment and activation of STAT5 and the transcription of STAT5-target genes, such as *Bcl2*, *GzmB*, *Idb2*, *Mcl1*, *Pim2*, and *Prf1*, all of which are required for proliferation, survival, and cytotoxicity (58). Beyond the JAK/STAT pathway, γc family cytokines also function through other pathways, such as the MAP kinase- and phosphoinositol 3-kinase-dependent pathways (59).

Peptide recognition boosts adaptive NK cell expansion and differentiation under the assistance of short-term addition of IL-12 and IL-18, even though this subset does not express IL12/IL18R, especially g^-^NK, although the exact mechanism is unclear. The combined stimuli skew the adaptive NK phenotype and even contribute to hypomethylation of *IFNG* CNS1. Additionally, a short stimulation of IL-12 plus IL-18 induces down-regulation of FcRγ and CD7 in NKG2C^+/-^ cells. Transcriptional levels of adaptive NK cell-related genes are also affected by the combination of pro-inflammatory signals plus peptide recognition, such as *NCR3*, *SH2DB1*, *ZBTB32*, *ZBTB16*, *ZBTB20*, *ITGAL*, and *CRTAM*. These signals drive sustained transcription of genes encoding activation and apoptosis markers, such as *HLA-DR*, *TNFRSF9*, *LAG3*, *CTLA4*, and *PDCD1*, as well as effector molecules, such as *IL8*, *CSF2*, *IL10*, *GZMB*, *IFNG*, *TNF*, *CCL3*, *CCL4*, and *TNFSF10* (14).

## 4 Differentiation and Tissue Distribution of Adaptive NK Cells

**Figure 3:**
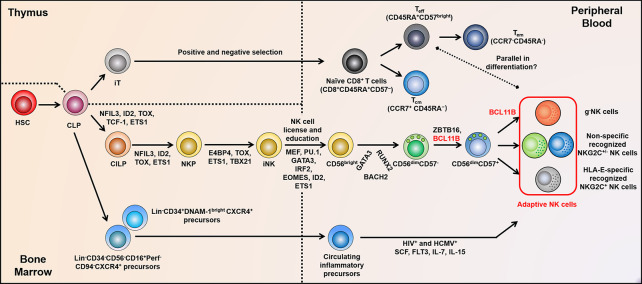
Adaptive NK cell development and differentiation. The positive and negative selection experienced by T cells in the thymus may be similar to the “license” and “education” of NK cells in BM. Subsequently, non-self KIRs expressed on NK cell surface facilitate adaptive NK cell diversity. TF networks show reciprocal regulation during distinct stages of NK cell differentiation, in particular, Bcl11b is fundamental for the differentiation of adaptive NK cells and c-NK cells. At the genome levels, adaptive NK cells accompanied by altered methylation properties are parallel to T_eff_ cells. In addition, the circulating inflammatory progenitor cells in PB may as a source of adaptive NK cells during HCMV and HIV co-infection. BM, bone marrow; KIRs, killer cell immunoglobulin-like receptors; TF, transcription factor; HSC, hematopoietic stem cell; CILP, common innate lymphoid progenitor; NKP, NK cell precursor; CLP, common lymphoid progenitor; iT, immature T cell; iNK, immature NK cell; c-NK, conventional NK; and PB, peripheral blood.

Generally, hNK cells account for approximately 5%–15% of the total PB lymphocytes. The half-life of circulating NK cells is approximately 14 days, and the proliferation is 4%–5% per day. hNK cells stem from a common innate lymphoid progenitor (CILP) *via* an NK cell precursor (NKP), both of which are differentiated from a common lymphoid progenitor (CLP) (60). Subsequently, under the regulation of TFs, such as TOX, TBX21, ETS1, and E4BP4, NKP further differentiates into immature NK cells (iNK) in an Eomes-dependent manner to become highly proliferative CD56^bright^ NK cells. Additionally, RUNX2, GATA3, and BACH2 constitute a regulatory network that dominates the formation of the mature CD56^dim^ subgroup. Besides the down-regulation of CD56, terminal differentiation of NK cells involves the modulation of receptor profile, the acquisition of cytotoxic function, and even epigenetic modifications, which constitute the heterogeneity of NK cells (61). Although the differentiation of adaptive NK cells is poorly understood, it is feasible that adaptive NK cells may be derived from the CD56^dim^CD57^+^ subgroup. Holmes et al. considered that the TF regulation network shifts with the down-regulation of *ZBTB16* and the up-regulation of *BCL11B*, which may be mutually antagonistic. Ultimately, *BCL11B* acts as the top of the pyramid to regulate *YBX3*, *PBX4*, *SATB2*, *IRF4*, and *NFIC*, in the formation of adaptive NK cells (62) (**Figure 3**).

Mounting evidence has shown that the development of adaptive NK cells and CD8^+^ T cells has an analogous process. Transcriptional profiling reveals that the differentiation axis of CD7^lo^NKG2C^+^NKp30^lo^CD57^+^ adaptive NK cells converges toward that of CD45RA^hi^CD28^-^CCR7^-^CD27^-^CD57^+^CD8^+^ T cells (62). Immature T cells complete positive and negative selection to obtain MHC restriction and central immune tolerance, respectively, in the thymus. Correspondingly, iNK cells undergo a similar process known as “missing self” and “education” or “license” by expressing self- and non-self KIRs to form a repertoire that binds to MHC-I ligands. Eventually, mature NK cells are formed with effector function and self-tolerance (63–65). Kim et al. and Bryceson et al. further suggested that HCMV-induced epigenetic modification and Ab-dependent expansion are mechanisms underlying the formation of this memory-like NK cell pool, paralleling with cytotoxic effector T cell (T_eff_) differentiation (18, 29). The genome-wide analysis illustrated that the methylation characteristic of early mature NK cells (CD56^dim^CD57^bright^EAT2^+^) was different from that of T_eff_ cells. However, CD56^dim^CD57^bright^EAT2^-^ adaptive NK cells are quite similar to those of T_eff_ cells. Comparing adaptive NK cells with T_eff_ cells, 61 differential methylation regions are found; nevertheless, there are up to 2372 differences between early mature NK (CD56^dim^CD57^-^) and adaptive NK cells (18). Heath et al. suggested that the extensive expansion of NKG2C^+^CD57^+^ NK cells after sensing stress served to keep pace with HCMV reactivation or other factors behind such expansion; this adaptive property is similar to that of virus-specific CD8^+^ T cells in that it does not involve replication. The accumulation of this NK subset may reflect the NK immune response, which is homologous with the expanded adaptive T cell response, marked by the accumulation of HCMV-specific effector memory T cells (T_em_) (66). The integrative-omics analysis revealed that super-enhancers related to gene cohorts might coordinate NK cell function and localization. The TCF7-LEF1-MYC module in CD56^bright^ NK cells may participate in preserving the progenitor cells, which can further replenish adaptive cells after BLIMP1 induction. TCF7-LEF1 is weakly expressed in adaptive NK cells that show a terminal differentiation state closer to that of T_em_ and Th1 cells (67). These results further illustrate the correlation between adaptive NK cells and T_eff_ during differentiation and tissue colonization (**Figure 3**).

GATA-2 expression is confined to hematopoietic stem and progenitor cells (HSPC). Patients with heterozygous *GATA2* mutation frequently exhibit immunodeficiency, such as no NK cell progenitors. However, in some patients with *GATA-2* mutation, a durable survival or self-renewing PLZF^-^ adaptive NK cell pool has been observed. These cells express low levels of perforin and GZMA but high levels of GZMB. Similar to adaptive NK cells in healthy individuals, these NK cells respond to FcR pathway-stimulation but not to IL-12 plus IL-18 (68). The expression of cytotoxic granule components is controlled by T-bet and Eomes (69). Consistently, T-bet expression in these adaptive NK cells is significantly lower than that in typical PLZF^+^ NK cells, but there is no clear difference in Eomes expression. PLZF^-^ adaptive NK cells in patients with *GATA2* mutation show functional properties related to those of adaptive NK cells from healthy individuals. Although they help to kill infected cells, the high-frequency adaptive NK cells in individuals with *GATA2* mutation may cause inflammation-driven BM failure (68). Another important discovery suggested that the differentiation and function of adaptive NK cells can be independent of glycosylphosphatidylinositol (GPI) anchors in patients with paroxysmal nocturnal hemoglobinuria (PNH), which frequently undergoes somatic X-linked *PIGA* mutations that lead to a lack of GPI anchored membrane proteins on hematopoietic cells (70). In short, the homeostasis of adaptive NK cells in the periphery may not depend on HSPC or CD56^bright^ precursor NK cells.

A recent study showed that in HIV^+^ anti-retroviral-treated and HCMV^+^ reactivated patients, HCMV-controlling NKG2C^+^ adaptive NK cells may be derived from novel circulating inflammatory precursors marked by Lin^-^CD34^+^ DNAM-1^bright^CXCR4^+^ and can rapidly differentiate into an NKG2C^+^KIR^+^CD57^+^ NK subset. Moreover, other Lin^-^CD34^-^CD56^-^CD16^+^Perf^-^CD94^-^CXCR4^+^ precursors from CLP also possess the potential to develop toward memory-like NKG2C^+^ NK cells (71) (**Figure 3**). It is inferred that the precursors of adaptive NK cells are released and circulate in PB during viral infection, where they exert powerful killing activity upon maturation. Overall, to date, it is unclear whether adaptive NK cells are specifically differentiated from a subgroup of CLP. No current studies have reported that c-NK cells from HCMV seronegative donors can be induced to differentiate into an adaptive NK cell state *in vitro* (72). Existing studies have shown that they are more likely to be NK cells with cytotoxic T-like specificity. Thus, the concrete mechanisms of their differentiation source and regulatory factors require further experimental data support.

After maturation, adaptive NK cells migrate to multiple peripheral organs but are preferentially present in non-lymphoid organs. In addition to PB, adaptive NK cells can colonize the tonsils, lymph nodes (LNs), liver, pleural fluid, and other sites. Interestingly, adaptive NK cells can also infiltrate into tumor tissues, such as in non-small cell lung cancer (NSCLC) (73) and colon cancer (unpublished data). Additionally, Shah et al. explored the presence of NK cell memory in primates and discovered a systematic distribution of Δg NK cells, referred to as g^-^NK, with adaptive features through a rhesus macaque model. They also found that in addition to PB, Δg NK cells were also distributed in the spleen, BM, multiple lymph nodes, and colon mucosa. Apart from CMV-induced expansion, rhesus cytomegalovirus (RhCMV)-primed Δg NK cells can also be affected by simian immunodeficiency virus (SIV) infection, following which they are recruited into the mucosa and effector tissues. Δg NK function is subverted by SIV infection through inhibition of the CD16-mediated CD3ζ-ZAP70 pathway. Most importantly, Δg NK cells chose CD3ζ-Zap70 signaling as an alternative pathway, which can modulate CD16 density, mucosal homing, and NK function while forgoing typical γ-chain/SYK signaling (74). This model provides an optimized experimental animal model for further exploration of the function of adaptive NK cells in the future.

## 5 Clinical Relevance of Adaptive NK Cells

The imprint mediated by HCMV infection on the adaptive NK cell repertoire is usually fixed at the initial encounter. The stability of the impact probably depends on the host and virus genetics, as well as environmental factors, such as age and viral load at initial infection. However, inefficient control of potential infections is associated with immunosenescence or dysregulated immunity, which may promote the expansion of pre-differentiated adaptive NK cells (75). In umbilical cord blood transplantation (UCBT) recipients, HCMV reactivation may induce rapid phenotypic reconfiguration, including the early and late acquisition of certain adaptive characteristics (76). Adaptive NK cells may play a part in in processes involving specific Abs, such as immune complex diseases or targeted Ab-based cancer therapy. As the definition of adaptive NK cells does not entirely overlap with that of the previously discovered NKG2C^+^ NK cells, it remains to be elucidated whether adaptive NK cells are also suitable for these models. Moreover, it is currently unclear whether adaptive NK cells may protect against or promote disease progression.

### 5.1 Viral Infection

HCMV impacts both innate and adaptive subsets and immune responses during its three-phase infection, that is, acute infection, persistence, and latency/reactivation, leading to immune system shaping (77). Early studies have shown that NK cells are critical for controlling herpesvirus infection (78). Not only are they involved in first-line innate defense, but the latest data based on mice show that memory-like NK cells with adaptive lymphocyte characteristics can be triggered in phase I of HCMV infection and can be preferentially maintained in the later phases. The presence of an abundance of adaptive NK cells may be associated with considerable clinical prognosis, especially for patients with cancer who use approved monoclonal antibodies (mAbs) that can effectively trigger ADCC. However, NKG2C^hi^ NK cells account for 50% of the entire compartment in some HCMV-infected individuals, which will reduce the diversity of the overall NK pool and attenuate surveillance of heterologous infection or tumor progress (77).

In virologically suppressed patients with HIV, expanded adaptive NK cells express low levels of HLA-DR and CD38 and lack NKp30 and NKp46 expression. This may be damaging to NK-mediated immune surveillance in patients receiving combination antiretroviral therapy. The presence of this subgroup is related to HCMV serology and soluble CXCL10 in plasma (25). Likewise, in cHBV and HCMV co-infected individuals, adaptive NK cells show increased frequency and polarization compared to c-NK cells (79). Furthermore, adaptive NK cells are also present in individuals with HCV infection. Notably, direct antiviral therapy can alter the PD-1 expression and ADCC activity of adaptive NK cells, resulting in improved effector function (23). Another interesting finding indicates that in chronic HCV-infected HCMV^+^ subjects, adaptive NK cells account for most of the CD56^neg^CD16^+^ population, in addition to constituting a fraction of CD56^dim^CD16^+^. Adaptive NK cell carriers have lower levels of liver enzymes and fibrosis, revealing that the involvement of adaptive NK cells in chronic HCV infection can effectively ease liver disease (80). Petitdemange et al. demonstrated that acute Chikungunya virus (CHIKV) infection promotes a transient change in the phenotype and function of NK cells, as evidenced by transient clonal expansion of NK cells co-expressing CD94/NKG2C and HLA-C1 alleles, decreased expression of NKp46, NKG2A, and CD161, and up-regulated expression of CD57, ILT2, and NKp44. Intriguingly, NKG2C can rapidly increase in response to acute CHIKV infection and enter the contraction phase following viral clearance. The clonal expansion of this subset is correlated with viral load, indicating that NK cells can sense CHIKV from the onset of infection, thereby helping to eliminate the virus (81). Hart et al. found that the frequency of adaptive NK cells is positively associated with a favorable prognosis of malaria after natural exposure to *Plasmodium falciparum*. Erythrocytes infected with *P. falciparum* can induce NK cell degranulation and be lysed *via* ADCC in the presence of plasma from patients infected with malaria. The significance of this research is that an imminent vaccine with IgG1 and IgG3 Abs against *P. falciparum* Ags expressed on the surface of infected erythrocytes could stimulate CD16 to ultimately achieve effective elimination of *P. falciparum* (82).

In the process of coronavirus disease 2019 (COVID-19), caused by severe acute respiratory syndrome coronavirus 2 (SARS-CoV-2), adaptive NK cells are enriched in patients compared to healthy donors. This difference is particularly obvious in patients with fatal outcomes. Moreover, the frequency of adaptive/memory NK cells in deceased patients increases statistically (83). Other work showed that adaptive NK cells had signs of expansion in patients with COVID-19, but this did not rely on the HCMV activation secondary to COVID-19. Compared to nonresponding adaptive NK cells, responding adaptive NK cells express HLA-DR, CD38, CD62L, and MIP-1, while the expression of NKG2A, NKG2D, TIGIT, and CD25 is marginal (84). Furthermore, NKG2C and HLA-E host genetic variation may determine the severity of COVID-19 symptoms. Indeed, the expression levels of *KLRC2* ^del^ and HLA-E*0101 are significantly higher in patients hospitalized with COVID19, especially in those requiring intensive care, compared to patients with mild symptoms (85).

### 5.2 Transplantation Immunity

As the first reconstituted lymphocyte after transplantation, NK cells may be strongly imprinted by HCMV, especially in a transplant setting where T-cell immune function is chronically impaired (86). Muccio et al. noticed that in UCBT recipients undergoing HCMV reactivation, the down-regulated expression of FcRγ was detected at a later time point (i.e., twelfth month), while other adaptive NK phenotypes were acquired early after UCBT (i.e., sixth month). Additionally, FcRγ was found to be more frequently down-regulated in NKG2C^-^CD57^+^ NK cells compared to the classic memory-like NKG2C^+^CD57^+^ subset. They further demonstrated that down-regulation of PLZF and FcRγ are independent events, as the down-regulation of FcRγ in NKG2C^-^CD57^+^ NK cells does not show a corresponding down-regulation of PLZF at 24 months post-transplantation. This may require HCMV reinfection/reactivation to achieve stable epigenetic reprogramming that allows for sustained FcRγ silencing. The expansion ability of adaptive NK cells is significantly improved in the presence of IgG Abs, suggesting that the low frequency of adaptive NK cells is related to the impaired humoral response in the UCBT recipient (76).

Whether the increased proportion of adaptive NK cells mentioned above (anti-COVID-19 immune response) is a double-edged sword for the body to defend against exogenous infection or tumor immunity remains a pressing research question. According to the existing results, the potential cross-reactivity of adaptive NK cells can be beneficial in the treatment of leukemia. Interestingly, patients with acute myeloid leukemia (AML) who undergo hematopoietic stem cell transplantation (HSCT) have a lower relapse rate and superior disease-free survival (DFS) when the donor and/or recipient is HCMV seropositive before transplantation; these individuals benefit from the expansion of CD56^dim^CD57^+^NKG2C^+^ adaptive NK cells in response to HCMV reactivation (87). Nguyen et al. considered that NKG2C^+^ adaptive NK cells can eradicate the minimal residual disease by cross-reactive recognition with HLA-E^+^ leukemic blasts (88). *Ex vivo* experiments have shown that adaptive NK cells enriched in HLA-E ligands have enhanced alloreactivity to HLA-mismatched targets and even serve as a specific and efficient killer of allogeneic pediatric T- and precursor B-cell acute lymphoblastic leukemia (ALL) blasts (89).

Immunosuppressed kidney transplant recipients (KTRs) induced by thymoglobulin have a poor immune response and may be infected after receiving transplants collected from HCMV seropositive donors. In KTRs receiving immunosuppressive therapy, primary HCMV infection, reactivation, or reinfection is associated with graft loss and reduced patient survival. The damaging effect of HCMV on KTRs can be prevented by post-transplant viremia screening, antiviral prophylaxis, and mTOR targeting drug treatment. However, the control of HCMV replication in KTRs depends on the recipients’ immune system to restrain the pathogen (90). Among KTRs that are clinically stable for more than 2 years after transplantation, HCMV seropositivity or HCMV DNA replication can alter the NK cell phenotype. In KTRs with active HCMV replication, an expanded FcRγ^–^LIR-1^+^NKG2C^–^ NK subset exhibits vigorous ADCC function in the presence of immobilized HCMV glycoprotein B reactive Abs; however, low expression of perforin after co-culture with K562 suggests that the natural killing ability of this subset is weakened. These results demonstrate that HCMV can relapse in asymptomatic KTRs and that this recurrence leads to continuous exposure of NK cells to HCMV to promote the expansion and persistence of adaptive NK cells *in vivo* (91). This expansion also applies to the involvement of NKG2C^+^ adaptive NK cells in the control of HCMV in KTRs. Consistently, the incidence of viremia after transplantation is reduced in the case of transplantation of large numbers of NKG2C^+^ NK cells (92).

### 5.3 Tumor Immunity

Based on preclinical and clinical observations, as well as the aforementioned HSCT, adaptive NK cells promote the control of hematopoietic malignancies and prevent recurrence of disease. An analysis of 215 patients with hematological malignancies demonstrated that transplantation conditions significantly affect the functional NK cell pool. The levels of adaptive NK cells in recipients receiving non-myeloablative therapy (NMAC) are statistically higher than those in recipients receiving myeloablative therapy (MAC), and high HCMV neutralizing Ab titers have certain promotion significance for adaptive NK cell expansion. Multivariate analysis of DFS, relapse, and treatment-related mortality (TRM) suggests that NMAC recipients with a large number of adaptive NK cells have favorable clinical outcomes 6 months after HCT (93).

Similar effects have been observed for the treatment of multiple myeloma (MM) with CD38-specific Ab, daratumumab. CD38 is highly expressed in hematopoietic stem cells (HSCs) and MM and functions as a receptor-mediated adhesion to regulate the cyclase and hydrolase activities. Daratumumab targeting CD38 can lyse lymphoma cells or CD38^+^ immunosuppressive cells through Fc-mediated complement-dependent cytotoxicity (CDC), ADCC, and Ab-dependent cellular phagocytosis (ADCP) (94). Immunophenotypic characteristics and functional analysis of adaptive NK cells from newly diagnosed multiple myeloma (NDMM) patients showed that adaptive NK cells exhibit an observably lower level of CD38 expression compared to c-NK cells, suggesting that they can evade daratumumab-induced fratricide. A recent study has shown that knockdown of the ectoenzyme CD38 in high-affinity non-cleavable variant of CD16a (hnCD16a)-induced pluripotent stem cell (iPSC) NK cells enhances their metabolic capacity, particularly glycolysis and cysteine metabolism, and improves their relative resistance to oxidative stress. The metabolic profile of this subset is also observed in adaptive NK cells (95). Encouragingly, CD38^low^ adaptive NK cells exert a powerful daratumumab-mediated ADCC *in vitro*, and the frequency of this subset is positively correlated with the effector function of daratumumab (96). *In vivo* or *in vitro* studies by Bigley et al. further support the effective anti-tumor effect of g^-^NK cells combined with therapeutic mAbs, daratumumab and elotuzumab, targeting signaling lymphocytic activation molecule F7 (SLAMF7), in MM. Consistent with the above, g^-^NK cells express minimal levels of CD38 and SLAMF7 on their surface. In NSG mice, following *in vitro* expansion, g^-^NK and c-NK cells were adoptively transferred and supplemented by daratumumab and rhIL-15 to achieve *in vivo* expansion. After 31 days, g^-^NK cells persisted in the blood and spleen, with more than 10-fold higher numbers than c-NK cells. In a disseminated orthotopic xenograft MM.1S model, compared to daratumumab plus c-NK cells, adoptive treatment with a combination of daratumumab and g^-^NK cells reduces average tumor burden by > 99.9%. Moreover, 5 of the 7 mice eliminated the burden of myeloma, with a survival rate of up to 100% after 60 days. Interestingly, the persistence of expanded g^-^NK cells is detected in the blood, spleen and, BM relative to that of c-NK cells in the MM model (57).

Contrary to the positive role played by adaptive NK cells in hematological tumors, HCMV infection and the existence of adaptive NK cells in solid tumors may be a negative factor. A solid tumor is a major adverse outcome of orthotopic liver transplantation (OLT), and 60%–90% of transplant recipients develop HCMV infection due to reactivation of the latent virus or new infection after long-term immunosuppressive treatment. HCMV can infect tumor cells, including medulloblastoma and colon carcinoma, and HCMV infection of tumor cells may disrupt the recognition by NK cells *via* down-regulating the expression of MHC class I molecules on tumor cells (97–99). Baryawno et al. discovered that HCMV infection of primary medulloblastomas and medulloblastoma cell lines further up-regulated COX-2 expression and PGE_2_ production in tumors, thereby stimulating tumor cell proliferation (100). The work by Achour et al. emphasized the complexity of the NK cell response and its clinical impact after OLT. Importantly, the new development of head and neck neoplasm or colon cancer was associated with the aberrant expansion of adaptive NK cells and robust production of TNF-α in HCMV^+^ patients. In contrast, NK cells from patients with genitourinary system tumors had classic iNK cell characteristics, including high expression of NKG2A and powerful IFN-γ production. TNF-α is closely related to the replication of CMV and can significantly increase malignant transformation by triggering the NF-κB transcription activator, eventually leading to immune failure in controlling various malignant tumors. Nevertheless, the level of IFN-γ can predict the long-term survival rate of patients with gastrointestinal stromal tumors after treatment with imatinib mesylate (101). In summary, in an immunosuppressive environment, the interaction between the NK repertoire and HCMV status may greatly hinder the spectrum of immune surveillance, which is preferential to the growth and development of specific neoplastic tumors after OLT.

## 6 Therapeutic Potential of Adaptive NK Cells

NK cell-based immunotherapy demonstrates promise. Currently, NK cell strategies for tumor immunotherapy include autologous or allogeneic NK cell therapy activated *in vitro*, a combination of NK cells and mAbs, such as immune checkpoint inhibitors (ICIs), rituximab, daratumumab, trastuzumab, and cetuximab, and construction of CAR-NK cells (102). Cytokine-induced human memory-like NK cells in NSG mice reveal an enhanced response to stimuli from several weeks to months after the initial adoptive transfer and a superior anti-tumor activity against AML. Similarly, in a first-in-human phase I clinical trial, adoptively transferred memory-like NK cells proliferate and expand in patients with AML and manifest a robust response to leukemia target (103). Chimeric Ag receptors (CARs) have been applied to improve the specific recognition of tumor cells by effector lymphocytes. CD19-CAR-memory-like NK cells targeting CD19 have shown a promising anti-tumor response to lymphoma in a preclinical study (104). For a long time, NK cells have been considered as short-lived innate effector cells (105). However, several lines of evidence indicate that adaptive NK cells have an unexpected long lifespan compared to c-NK cells, resembling adaptive memory cells, which can live for months to years (16). Based on the success of cytokine-induced memory-like NK and g^-^NK cells coupled with daratumumab *in vivo* and the durable longevity of adaptive NK cells, we hold the opinion that adaptive NK cells are suitable candidates for adoptive cell therapy.

**Figure 4:**
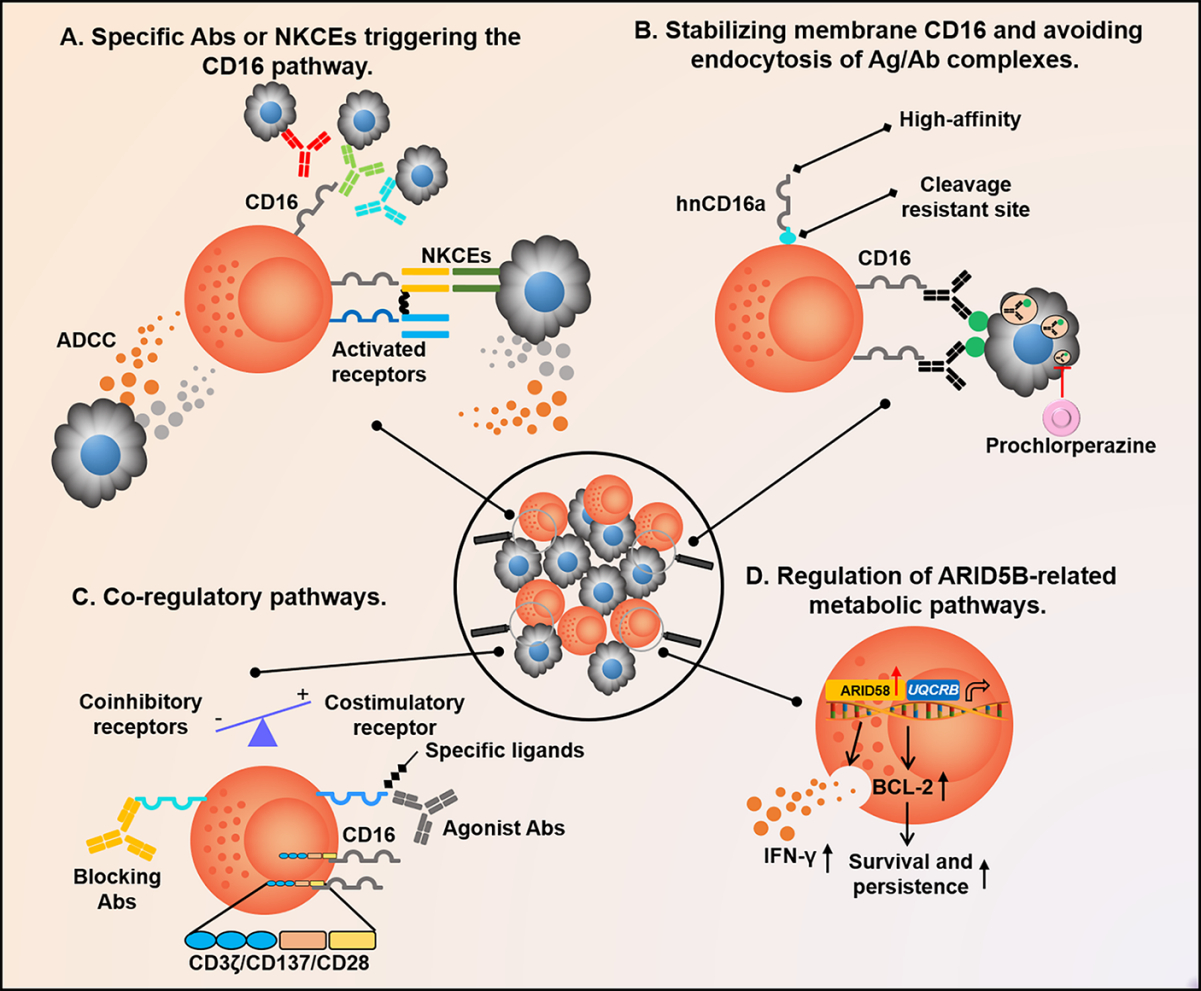
Future therapeutic strategies are based on adaptive NK cells. **(A)** When the CD16 pathway is activated by Abs or NKCEs binding to different targets, adaptive NK cells secrete numerous IFN-γ and TNF-α to regulate anti-tumor immunity; **(B)** Methods to significantly improve the efficiency of ADCC can be considered from two aspects of adaptive NK cells and target cells, such as avoiding CD16 on the surface of adaptive NK cells being cleaved by ADAM17 or MMP, enhancing its binding with Fc segment in a high-affinity manner, and advancing Ag presentation to the surface of tumor cells to reduce endocytosis; **(C)** Adding 4-1BB/CD28 and CD3ζ co-stimulatory signals to adaptive NK cells, or blocking the co-inhibitory pathway with the antagonistic Ab EOS-448 of TIGIT, and stimulating the co-activation pathway of CD2, NKG2C, and 4-1BB with agonist are all effective means to strengthen adaptive NK cell function; **(D)** Up-regulation of ARID5B expressed in adaptive NK cells can alter its metabolic characteristics, resulting in longer persistence and increased function. NKCEs, NK cell engagers; ADCC, Ab-dependent cell-mediated cytotoxicity; ADAM17, A disintegrin and metalloproteinase-17; and MMP, Matrix Metalloproteinase.

Here, we propose several aspects to accelerate the clinical application of adaptive NK cells:

1) Developing Ag-specific Abs or NK cell engagers (NKCEs) that trigger the CD16 pathway to mimic TCR or BCR and significantly broadens the spectrum of Ag specificity of adaptive NK cells (106–108).

2) Stabilizing membrane CD16 and target Ag. Methods to significantly improve ADCC efficiency can be considered in terms of both adaptive NK and target cells, such as avoiding CD16 on the surface of adaptive NK cells being cleaved by A disintegrin and metalloproteinase-17 (ADAM17) and Matrix Metalloproteinase (MMP), enhancing its high-affinity binding to the Fc segment, or advancing of Ag presentation to the surface of tumor cells to reduce endocytosis (109, 110).

3) Manipulating co-regulatory pathways expressed on NK cells with either co-activated receptor agonist or co-inhibitory receptor blocking Abs, all of which are effective means to strengthen the activation and functions of adaptive NK cells, such as adding 4-1BB/CD28 and CD3ζ co-stimulatory signals, blocking the co-inhibitory pathway with EOS-448 against TIGIT, and stimulating the co-activation pathway of CD2, NKG2C, and 4-1BB (111).

4) Up-regulating ARID5B in adaptive NK cells to maintain their long-term survival. ARID5B is a transcriptional regulator that regulates anti-apoptosis, oxidative metabolism, and IFN-γ secretion in adaptive NK cells (56) (**Figure 4**).

Our current results show that lentivirus infection *in vitro* can lead to down-regulation of FcRγ in c-NK cells, resulting in a prominent increase in the proportion of adaptive NK cells (unpublished data). Therefore, in the future, immunotherapy based on the above strategy will also apply to general NK cells or non-HCMV reactive memory-like NK cells. HCMV reactive adaptive NK cells have a unique advantage of being invoked as a cell therapy tool. They have a stronger Ab-dependent response-ability than c-NK cells and can maintain a longer survival time *in vivo*, which greatly exerts ADCC function and improves the curative effect.

## 7 Conclusion

A primary physiological role of NK cells is to provide a primary defense against pathogenic organisms during the initial response period when the adaptive immune system is activated. Although NK cells respond to various microorganisms, including bacteria and protozoa, they are particularly imperative in viral infections. Through Fc receptor-mediated recognition of target cells bound by Abs, adaptive NK cells can produce a robust response to infected cells, especially during chronic or recurrent infection, where reactive Abs are readily available. Considering that adaptive NK cells are present in a fraction of healthy individuals, the presence or absence and frequency of adaptive NK cells may accelerate immune heterogeneity among individuals against infection and cancer. Adaptive NK cells can serve as a novel tool for the clinical treatment of chronic diseases, such as malignancies and viral infections.

## Author Contributions

TZ and FG took the lead in structuring and writing the manuscript, ZZ, YL, GS, and GY helped modify the manuscript. All authors contributed to the article and approved the submitted version.

## Funding

This research was funded by the Key Project of Hunan Province (No. 2022WK2012).

## Conflict of Interest

The authors declare that the research was conducted in the absence of any commercial or financial relationships that could be construed as a potential conflict of interest.

## Publisher’s Note

All claims expressed in this article are solely those of the authors and do not necessarily represent those of their affiliated organizations, or those of the publisher, the editors and the reviewers. Any product that may be evaluated in this article, or claim that may be made by its manufacturer, is not guaranteed or endorsed by the publisher.
